# Tuberculose pelvi-péritoneale pseudotumorale: à propos de quatre cas

**Published:** 2012-11-15

**Authors:** Hanane Saadi, Nissrine Mamouni, Sanaa Errarhay, Chahrazed Bouchikhi, Abdelaziz Banani, Hicham Ammor, Nadia Sqalli, Siham Tizniti, Karim Benmajdoube, Khalid Maazaze, Hind Fatmi, Afaf Amarti

**Affiliations:** 1 Service de gynécologie obstétrique I CHU Hassan II Fès Maroc; 2 Service de radiologie CHU Hassan II Fès Maroc; 3 Service de chirurgie viscérale, CHU Hassan II Fès Maroc; 4 Service d'anatomopathologie CHU Hassan II Fès Maroc

**Keywords:** Tuberculose pseudo tumorale, pelvienne, radiologie, traitement, pronostic, pseudo tumor tuberculosis, pelvic, radiology, treatment, prognosis

## Abstract

La tuberculose pelvienne pseudo tumorale est une maladie infectieuse curable. Son tableau clinique est souvent trompeur simulant une tumeur ovarienne ou tubaire. Le but de notre travail est de préciser les caractéristiques cliniques, biologiques et radiologiques de cette pathologie et sa prise en charge. Nous rapportons une étude rétrospective à propos de quatre observations. L’âge moyen de nos patientes est de 24 ans (16 ans, 40 ans), trois parmi elles étaient célibataires. Le motif de consultation est dominé par les douleurs abdominopelviennes chroniques. Les résultats des explorations radiologiques (échographie pelvienne associé à la TDM ou IRM pelvienne) ont été en faveur d'une tumeur ovarienne dans trois cas et d'un hydrosapinx bilatéral pour un cas. L'ascite a été présente dans tous les cas. Le dosage de la Ca 125 a été élevé. La prise en charge a été l'exploration chirurgicale soit par c'lioscopie ou laparotomie. Deux cas ont bénéficié seulement des biopsies et deux patientes ont eu une salpingectomie bilatérale devant l'aspect pseudo tumoral très suspect. L’étude histologique a confirmé des lésions graulomateuses avec nécrose caséeuse. Le traitement par les antibacillaires a été instauré selon le protocole 2ERHZ/ 4RH. La tuberculose pelvienne pseudo tumorale est l'apanage de la femme jeune. Son pronostic est lié à l'infertilité séquellaire.

## Introduction

La tuberculose est une maladie infectieuse curable. Sa prévalence a connu une recrudescence dans le monde entier ceci s′explique par l′immunodépression liée à l′infection par le VIH [1]. Sa localisation pelvienne représente 6 à 10% [2] dominée par l′atteinte tubaire, puis cervicale et endométriale. Le tableau clinique ainsi que radiologique et biologique dans sa forme pseudotumorale simulant en général une tumeur ovarienne maligne.

## Patients et observations

### Observation 1

Mme G.Z, âgée de 40 ans, paucipare, sans antécédents pathologiques notables, adressée du service de gastrologie entérologie pour prise en charge d'une ascite de moyenne abondance.

**Figure 1:**
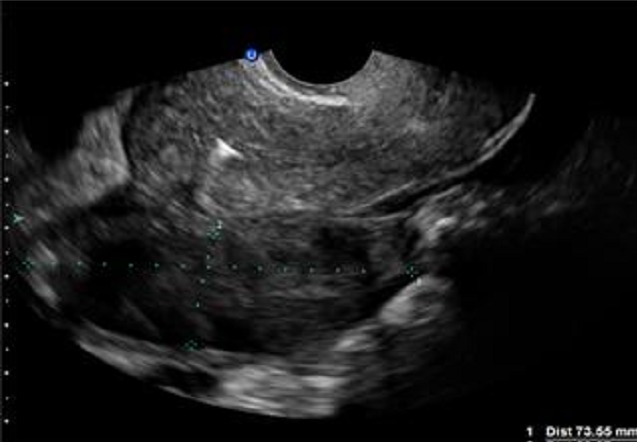
Échographie endovaginale montre une image rétroutèrine hétérogène, échogène tissulaire ne prenant pas le doppler couleur

Le début de la symptomatologie remonte à 3 mois où la patiente a présenté des douleurs abdominales diffuses isolées. L′examen clinique a révélé une matité diffuse et la taille utérine a été difficilement appréciable. Le bilan biologique a révélé un taux de Ca 125 élevé avec un test à la tuberculine négative et la recherche de BK dans les crachats sur trois prélèvements a été négative. La radiographie thoracique a été normale. Une échographie abdominopelvienne a objectivé la présence d'une ascite de moyenne abondance, un utérus de taille normale une image en rétro utérine hétérogène, échogène, mal limitée par endroit, ne prenant pas le doppler couleur, les ovaires non vus (Figure 1). La tomodensitométrie abdominale a montré la présence de deux lésions tissulaires ovariennes bilatérales avec des adénopathies intra et rétropéritonèales ainsi qu′un épanchement intra péritonéal enkysté. La décision de laparotomie a été prise au cours de laquelle l'exploration a permis d'objectiver la présence d'une ascite cloisonnée faite de liquide séro hématique, un pelvis adhérentiel, avec présence d′une masse à surface irrégulière latéro et rétro utérine droite faisant 5cm, son ouverture accidentelle donne issu à un liquide blanchâtre prélevé. L'ovaire gauche a été vu mais l'ovaire controlatéral n'a pas été visualisé. L'examen extemporané de la coque de la collection est revenu en faveur de granulome inflammatoire avec nécrose caséeuse, ce qui a été confirmé à l'examen anatomopathologique définitif avec un résultat en faveur d'une paroi tubaire dont l’épithélium est hyperplasique et le chorion est siège d'une lésion granulomateuse de tuberculose ovarienne avec nécrose caséeuse. La biopsie des granulations a été en faveur de lésions granulomateuse et la cytologie du liquide péritonéal est en faveur d'un liquide inflammatoire. La patiente a été déclarée sortante trois jours après avec des suites opératoires simples, mise sous chimiothérapie antibacillaire à base de 2ERHZ/4RH. L′évolution a été bonne.

### Observation 2

**Figure 2:**
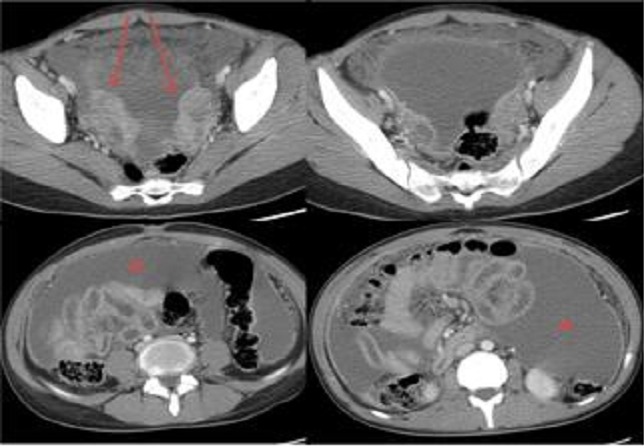
TDM abdomino-pelvienne, après injection du produit de contraste, coupes axiales objective deux masses solido-kystiques latéro-utérines (flèches), associées à une ascite encapsulante(étoile) avec épaississement et rehaussement péritonéal, en faveur de tumeurs ovariennes avec carcinose péritonéale

**Figure 3:**
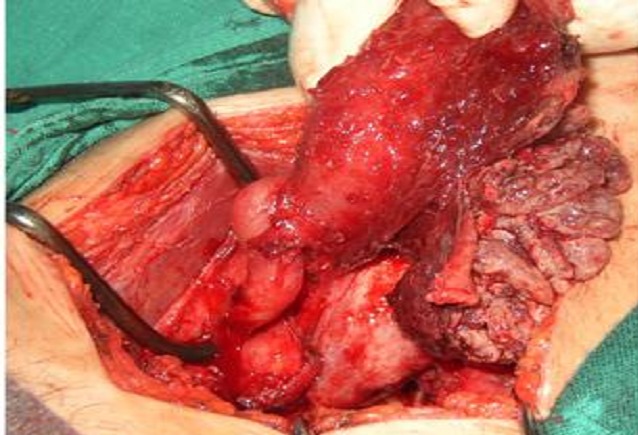
Vue opératoire d'une laparotomie d'une tuberculose tubaire et péritonéale

Mlle CM, âgée de 18 ans, célibataire et sans antécédents pathologiques notables. Admise pour prise en charge de douleurs abdomino-pelviennes chroniques chez qui l'examen clinique a objectivé une matité abdominale diffuse. L′échographie abdomino-pelvienne a révélé une ascite de grande abondance cloisonnée, l'utérus de taille normale et l'ovaire droit est augmenté de taille 50mm de grand axe et vascularisé. La tomodensitométrie a montré deux masses solido-kystiques latéro-utérines, associées à une ascite encapsulante avec épaississement et rehaussement péritonéal, en faveur de tumeurs ovariennes avec carcinose péritonéale (Figure 2). Le taux de CA125 a été élevé (1766 UI/l) et la recherche des BK dans les crachats est négative. La laparotomie découvre une ascite cloisonnée avec gâteau péritonéal dont l'examen extemporané revenant en faveur d'un remaniement inflammatoire. La patiente a bénéficié d'une salpingectomie bilatérale devant l'aspect suspect des trompes: trompes très boudinées, tortueuses et dures (Figure 3). L'examen anatomopathologique révélant une tuberculose tubaire et péritonéale. La patiente a reçu une chimiothérapie antibacillaire selon le protocole 2ERHZ/4RH.

### Observation 3

**Figure 4:**
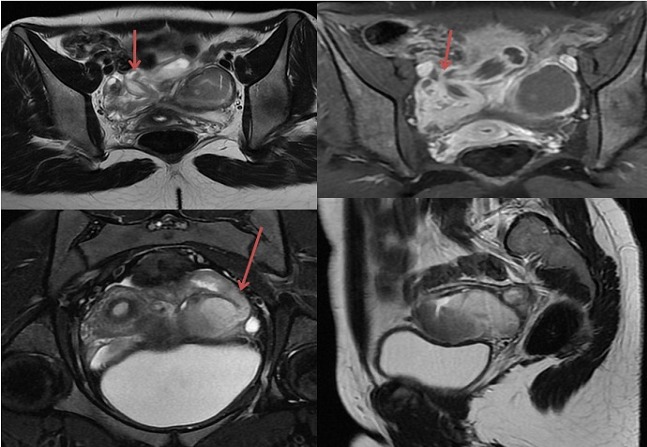
IRM pelvienne: hydrosalpinx bilatéral à contenu en hyposignal T2 et à parois rehaussée après contraste

Mlle NR, âgée de 16 ans, célibataire sans antécédents pathologiques notables, admise pour prise en charge de douleurs pelviennes majorées au niveau de la fosse iliaque droite, sans autres signes accompagnateurs digestif ou urinaire. Le tout évoluant dans un contexte d'asthénie et d'amaigrissement non chiffré. L'examen clinique trouve une légère sensibilité de la fosse iliaque droite chez une patiente apyrétique. Une échographie pelvienne a objectivé un utérus de taille normale avec deux images anéchogènes oblongues latéro utérines faisant 7cm à droite et 6 cm à gauche. Une IRM pelvienne a révélé la présence de 2 formations liquidiennes latéro utérines bilatérales de forme tubulé à paroi épaissie rehaussée après injection et contenant des cloisons incomplètes faisant évoquer un hydrosalpinx bilatéral. L'utérus et les ovaires sont d'aspect normal avec épanchement intra péritonéal de faible abondance (Figure 4). La décision de réalisation d'une exploration c'lioscopique, au cours de laquelle les deux trompes sont boudinées tortueuse, des ovaires difficile à explorer avec présence de granulations péritonéales diffuses. La décision de réaliser une laparotomie au cours de laquelle nous avons réalisé une salpingectomie droite et annexectomie gauche (l'ovaire étant adhèrent à la trompe).le résultat anatomopathologique est en faveur d'une tuberculose caseofolliculaire au niveau des trompes, de l'ovaire gauche et du péritoine. La patiente a été déclarée sortante après trois jours sous traitement antibacillaire 2ERHZ/4RH. L′évolution clinique a été favorable.

### Observation 4

**Figure 5:**
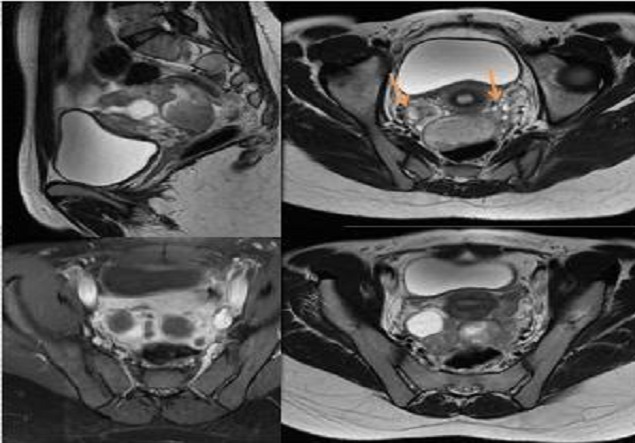
IRM pelvienne: Hydrosalpinx bilatéral à contenu en hyposignal T2, non rehaussé après contraste. Noter la visualisation d'ovaires normaux (flèches orange)

Mlle I.M de 22ans, célibataire, sans antécédent pathologique notable, hospitalisée pour prise en charge de douleurs pelviennes chroniques centrales atypiques sur un terrain d'apyrexie et d'amaigrissement non chiffré. L'examen gynécologique n'a pas pu être fait vu que la patiente a été vierge. La taille de l'utérus a été difficile à apprécier à travers le toucher rectal. L'examen abdominal objective la présence d'une matité des flancs. L’échographie pelvienne a montré un utérus de taille normal, l'ovaire droit est le siège d'une image hypoéchogène faisant 48,8mm de grand axe, l'ovaire gauche non vu, avec un épanchement intra péritonéal de moyenne abondance. Une imagerie par résonance magnétique pelvienne a révélé un Hydrosalpinx bilatéral à contenu en hyposignal T2, non rehaussé après injection du produit de contraste, les ovaires sont normaux (Figure 5). La patiente a bénéficié d'une c'lioscopie diagnostique objectivant la présence de multiples adhérences lâches, un pelvis adhérentiel, présence d'une lame d'ascite faite de liquide jaune citrin prélevée pour étude cytologique. L'utérus a été de taille normale, présence d'un hydrosalpinx bilatéral, l'ovaire droit augmenté de taille faisant 6 cm. La patiente a bénéficié de biopsies multiples, dont les résultats anatomopathologiques sont en faveur d'une lésion granulomateuse sans nécrose caséeuse au niveau des ovaires et du péritoine.

**Figure 6:**
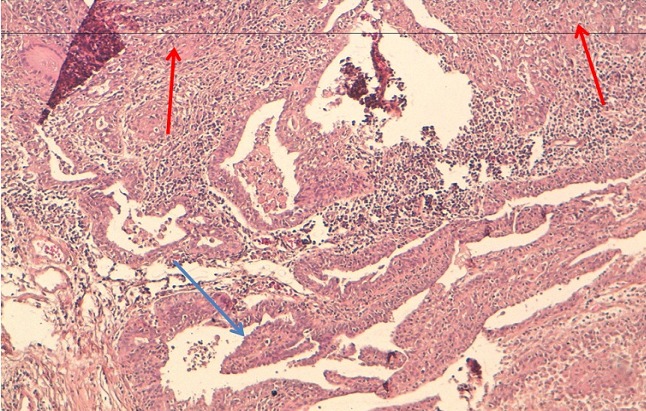
HES X 10: granulomes épithélioides et giganto-cellulaires (flèches bleus) avec une ébauche de nécrose caséeuse. Épithélium tubaire (flèche rouge)

La patiente fut ré hospitalisé quatre mois après, pour distension abdominale, chez qui l'examen clinique trouve une matité généralisée de tout l′abdomen. L’échographie pelvienne objectivant une ascite de grande abondance, un utérus de taille normale, présence en latéro utérin droit d'une image hétérogène faisant 42/37 mm non vascularisée au doppler d'où la décision d'une laparotomie exploratrice qui a permis d'objectiver un épanchement intra péritonéal de grande abondance avec liquide jaune citrin aspiré d'environ 3litres: prélèvement fait pour étude cytologique. La découverte de granulations blanchâtres en tête d’épingle au niveau de la vessie et le tube digestif. Présence d'un kyste au dépend de l'ovaire droit faisant 3cm dont le contenu a été aspiré. Les biopsies réalisées au niveau du péritoine pariétal et l′ovaire droit sont revenues en faveur de lésions granulomateuses avec nécrose caséeuse (Figure 6). La patiente a été mise sous chimiothérapie antibacillaire sous le schéma 2ERHZ/ 4RH. L′évolution à court terme est bonne.

## Discussion

La forme tumorale de la tuberculose génitale représente 15% de l′ensemble des localisations pelviennes de la tuberculose. Elle peut toucher toutes les femmes avec une prédominance les jeunes femmes entre 20 et 30 ans [3, 4].

L′agent pathogène est principalement le *Mycobacterium tuberculosis* ou le bacille de Koch secondairement le *Mycobacterium bovis*. Il s′agit d′un bacille acido-alcoolo-résistant à croissance lente (temps de dédoublement est de 15à 20 heures), ce qui explique l′évolution lente de la maladie [5]. L′atteinte urogénitale peut être contemporaine ou à distance à la primo-infection tuberculeuse. Sa localisation pelvienne se fait essentiellement par voie hématogène [6].

Sur le plan clinique, la symptomatologie est très variée et peu spécifique pouvant orienter à tort à une tumeur maligne de l′ovaire. En effet, les douleurs pelviennes, les masses pelviennes, l′ascite et l′amaigrissement peuvent être présent dans les deux pathologies. La recherche d′autres signes à type: troubles menstruels (dysménorrhées, aménorrhée), troubles digestifs, des signes urinaire qui sont inconstants. Par ailleurs, une infertilité peut être révélatrice dans 5 à 10% [7]. Une association avec d′autres localisations notamment pulmonaire ou digestive est à rechercher, mais leur absence est constatée dans 30 à 50% des cas [8].

Sur le plan radiologique, les données de l′échographie, du scanner et de l′imagerie par résonance magnétique ne sont pas spécifiques. La présence d′une masse pelvienne hétérogène à double composante, associé à une ascite, à un épaississement et rehaussement péritonéal est en faveur de tumeur ovarienne avec carcinose péritonéale. Parfois on peut avoir un aspect d′un hydrosalpinx bilatéral avec un ovaire augmenté de taille associé à une ascite. La lésion peut infiltrer la graisse de voisinage voire même l′envahir avec fistulisation aux organes de voisinage notamment le rectum [9].

L′augmentation du taux du marqueur CA 125 est retrouvée dans 80% des cancers ovariens. Néanmoins son taux peut être élevé dans des conditions normales (menstruation, grossesse) ou au cours des affections inflammatoires chroniques (tuberculose). Par conséquent, son dosage n′est pas un élément déterminant pour différencier une tuberculose pelvienne des cancers ovariens. Par contre, sa valeur réside surtout pour la surveillance des patientes sous antibacillaire [8].

L′exploration chirurgicale s′impose devant la forte suspicion d′une tumeur maligne. La voie d′abord peut être soit une laparotomie ou une laparoscopie. Cette dernière pose le diagnostic dans 97% des cas [10]. Ça n′a pas été le cas de notre quatrième observation où on a eu recours à la laparotomie devant la récidive de la symptomatologie.

Les biopsies transvaginales ou transabdominales échoguidées peuvent être proposées devant la forte suspicion de tuberculose [9, 11].

L′étude histologique de la biopsie oùde la pièce opératoire en montrant une lésion granulomatose gigantocellulaire avec une nécrose caséeuse spécifique du bacille de Koch pose le diagnostic da la tuberculose. L′étude bactériologique du liquide d′ascite est rarement positive [12].

Outre le cancer ovarien, il existe d′autres agents infectieux qui peuvent induire le même tableau clinique que la tuberculose ovarienne comme le *Streptococcus milleri*, les actinomycines ou les autres mycobactéries [4].

Le traitement de la tuberculose pelvienne est essentiellement médical (Table 1). Selon l'Organisation Mondiale de la Santé et l'American Thoracic Society recommandent un traitement pendant 6 mois reparti en une quadrithérapie intensif (isoniazide, rifampicine, Ethambutol, pyrazinamide) pendant deux mois, puis un traitement d'entretien pendant 4 mois par une bithérapie quotidienne (isoniazide, rifampicine) [13].

**Table 1:** Les antibacillaires de première ligne et leurs effets secondaires

Molécules	Durée	Posologie (adulte)	Mode d'action	Effets secondaires
**Isoniazide**	6mois	5mg/kg/jour En une prise	bactéricide	encéphalopathie, neuropathie distale, hépatotoxicité, ictère isolé, syndrome de Stevens johnson, fièvre, anémie hémolytique, neutropénie, lupus induit
**Rifampicine**	6mois	10mg/kg/j à jeun	bactéricide	inducteur enzymatique, Hépatoxicité Intolérance digestive, Anémie thrombopénie Immunoallergique Rash cutané, Coloration des urines et larmes en rouge
**Ethambutol**	2mois	15 à 20 mg/kg/j	bactériostatique	névrite optique rétro-bulbaire dose dépendante rash cutané neuropathie périphérique
**Pyrazinamide**	2mois	25mg/kg/j en une prise	bactéricide	Hyperuricémie asymptomatique Crise de goutte, hépatotoxicité, Polyarthralgies, Intolérance digestive, Rash cutané, Photosensibilité

La chirurgie est indiquée en cas de masse compressive ou fistulisée pour mettre à plat les cavités caséifiées [9]. Le pronostic de la tuberculose pelvienne est lié à l'infertilité des femmes jeunes. Le risque de l'infertilité tubo-ovarienne est estimé à 39% [8].

## Conclusion

La tuberculose pelvipéritonéale pseudotumorale est une pathologie infectieuse rare et curable. Sa symptomatologie clinique, radiologique et biologique simulant soit une tumeur ovarienne maligne soit un hydrosalpinx bilatéral associé à une ascite. Le pronostic est lié à l'infertilité tubo-ovarienne.
